# Addition of rapamycin and hydroxychloroquine to metronomic chemotherapy as a second line treatment results in high salvage rates for refractory metastatic solid tumors: a pilot safety and effectiveness analysis in a small patient cohort

**DOI:** 10.18632/oncotarget.3793

**Published:** 2015-04-12

**Authors:** Kwan-Hwa Chi, Hui-Ling Ko, Kai-Lin Yang, Cheng-Yen Lee, Mau-Shin Chi, Shang-Jyh Kao

**Affiliations:** ^1^ Department of Radiation Therapy and Oncology, Shin Kong Wu Ho-Su Memorial Hospital, Taipei, Taiwan; ^2^ School of Medicine and Institute of Biomedical Imaging and Radiological Sciences, National Yang-Ming University, Taipei, Taiwan; ^3^ Division of Chest Medicine, Shin Kong Wu Ho-Su Memorial Hospital, Taipei, Taiwan

**Keywords:** rapamycin, hydroxychloroquine, metronomic chemotherapy, autophagy

## Abstract

**BACKGROUND:**

Autophagy is an important oncotarget that can be modulated during anti-cancer therapy. Enhancing autophagy using chemotherapy and rapamycin (Rapa) treatment and then inhibiting it using hydroxychloroquine (HCQ) could synergistically improve therapy outcome in cancer patients. It is still unclear whether addition of Rapa and HCQ to chemotherapy could be used for reversing drug resistance.

**PATIENTS AND METHODS:**

Twenty-five stage IV cancer patients were identified. They had no clinical response to first-line metronomic chemotherapy; the patients were salvaged by adding an autophagy inducer (Rapa, 2 mg/day) and an autophagosome inhibitor (HCQ, 400 mg/day) to their current metronomic chemotherapy for at least 3 months. Patients included 4 prostate, 4 bladder, 4 lung, 4 breast, 2 colon, and 3 head and neck cancer patients as well as 4 sarcoma patients.

**RESULTS:**

Chemotherapy was administered for a total of 137 months. The median duration of chemotherapy cycles per patient was 4 months (95% confidence interval, 3–7 months). The overall response rate to this treatment was of 40%, with an 84% disease control rate. The most frequent and clinically significant toxicities were myelotoxicities. Grade ≥3 leucopenia occurred in 6 patients (24%), grade ≥3 thrombocytopenia in 8 (32%), and anemia in 3 (12%). None of them developed febrile neutropenia. Non-hematologic toxicities were fatigue (total 32%, with 1 patient developing grade 3 fatigue), diarrhea (total 20%, 1 patient developed grade 3 fatigue), reversible grade 3 cardiotoxicity (1 patient), and grade V liver toxicity from hepatitis B reactivation (1 patient).

**CONCLUSION:**

Our results of Rapa, HCQ and chemotherapy triplet combination suggest autophagy is a promising oncotarget and warrants further investigation in phase II studies.

## INTRODUCTION

Periodical delivery of standard recommended chemotherapy doses in some types of cancers is often associated with significant toxicity without therapeutic gain. The frequent administration of low doses (1/10^th^ to 1/3^rd^ of maximum tolerated dose, MTD) of certain anti-neoplastic drugs, known as metronomic chemotherapy, has demonstrated its efficacy and is now getting more popular [1]. The anti-cancer effect occurred principally via an anti-angiogenic/anti-vascular mechanism [1, 2]. Several in vivo experiments have shown that metronomic chemotherapy is more effective in combination with anti-angiogenic, immunotherapeutic, or targeted therapeutic agents [3, 4]. A growing number of clinical studies have adopted the concept of combined metronomic chemotherapy with anti-angiogenic therapy and have reported an increase in progression-free survival (PFS) in cases of recurrent glioblastoma multiforme [5], cisplatin-refractory ovarian cancer [6], advanced breast cancer [7, 8], non-small cell lung cancer [9], hepatoma [10], and colon cancer [11].

Autophagy is known to promote cancer growth and survival under conditions of nutrient deprivation, hypoxia, or DNA damage caused by chemotherapy [12]. Hydroxychloroquine (HCQ)-a clinically approved anti-rheumatoid drug-is an analogue of chloroquine (CQ) and acts as a lysosomotropic agent; HCQ inactivates lysosomal enzymes by increasing intralysosomal pH and significantly inhibiting the last process of autophagy [12]. A greater inhibition of the proliferative activity of various types of cancer has been reported when chemotherapy was combined with the inhibition of autophagy [13]. The premise of inhibiting autophagy to overcome chemotherapy resistance has been clinically investigated [14-19]. Rapa, a clinically approved anti-rejection drug, also known as mammalian target of rapamycin (mTOR) inhibitor, can induce cellular autophagy [20]. Autophagy modulation by combined treatment with an mTOR inhibitor (Rapa) and a lysosome inhibitor (HCQ) was shown to be effective in models of breast cancer, melanoma, and glioma [21-24]. We have found the triplet combination of HCQ, Rapa, and chemotherapy to be synergistic by pushing autophagy through Rapa + chemotherapy and then blocking the final autophagy process through HCQ [25].

A Rapa analogue, everolimus, in combination with HCQ, was found to inhibit growth of endothelial progenitor cells [26]. In this retrospective report, by collecting anecdotal cases in our institute, we found that addition of Rapa and HCQ to a metronomic chemotherapy therapy regimen might be an attractive way to increase sensitization to both the anti-cancer and the anti-angiogenesis effect of chemotherapy.

## RESULTS

### Patient characteristics

A total of 46 patients received metronomic chemotherapy from May 2012 to September 2014, and 25 of them fitting the study criteria were included in the analysis (17 women and 8 men). The median age was 62 years (range 47-76). The Eastern Cooperative Oncology Group performance status was 0 in 10 patients and 1 in 15 patients. The characteristics of the patient population are summarized in Table 1.

**Table 1:** Patient Demographics

Characteristics	Number
Total patients	25
Median age (years, range)	62 (47–76)
Sex	
Male	8
Female	17
Diagnosis	
Prostate cancerBladder cancer	44
Lung cancer	4
Colorectal cancer	2
Breast cancer	4
SarcomaHead and Neck cancer	43

### Tumor responses

**Figure 1:**
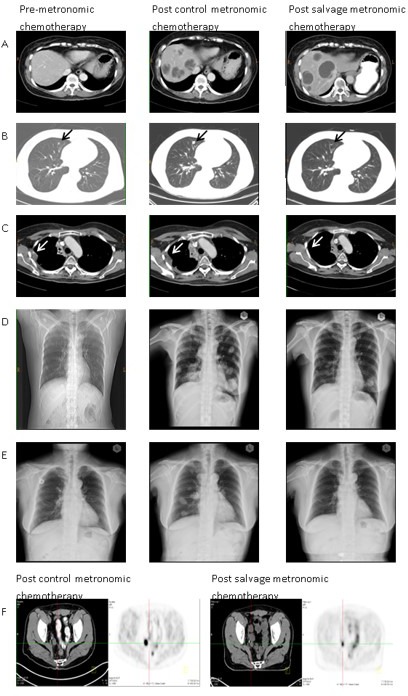
Illustrations of therapeutic responses in representative cases (**A**) patient #6, multiple liver metastases. Although the tumor did not show a reduction in size, the sum of diameters of enhancement regions were reduced by more than 30%, according to the modified RECIST criteria; (**B**) patient #8, right upper lung nodular lesion (black arrow); (**C**) patient #9, right pleural seeding tumor (white arrow); (**D**) patient #19, bilateral multiple lung metastases; (**E**) patient #20, bilateral multiple lung metastases; (**F**) patient #13, peritoneal seeding in the right pelvic region from positron emission tomography-computed tomography scans before and after salvage metronomic chemotherapy.

**Figure 2:**
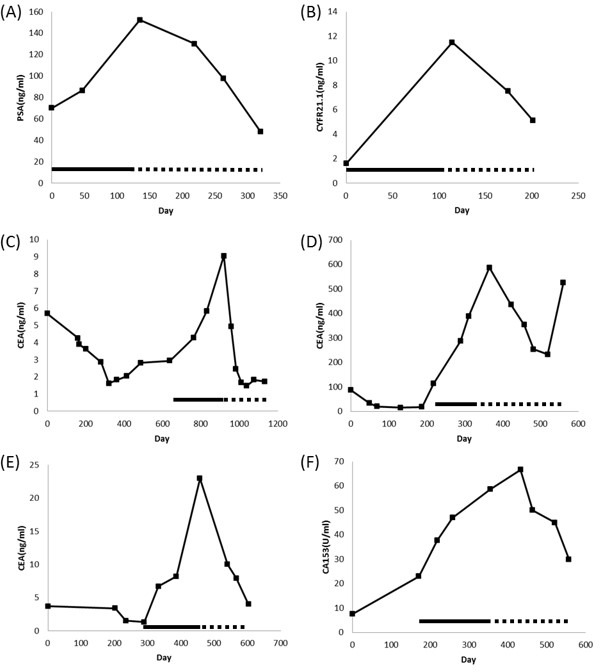
Representative plots of serum tumor markers versus time (**A**) patient #1; (**B**) patient #6; (**C**) patient #9; (**D**) patient #11; (**E**) patient #14; (**F**) patient #16.—Control metronomic chemotherapy; …… Rapa/HCQ + metronomic chemotherapy.

Within the group of 25 evaluable patients, 10 (40%) experienced PR, and 11 (44%) had SD. Eighty-four percent of patients experienced clinical benefits for more than 3 months. The clinical characteristics and results of patients who received this treatment strategy are summarized in Table 2. Representative images and tumor marker changes before metronomic chemotherapy, and before and after salvage metronomic chemotherapy are shown in Figures 1 and 2. Many patients documented in the study had non-measurable lesions but had a drop in tumor markers >50%. The median follow-up time was 11 months (range, 3–28 months). The median duration of salvage treatment was 4 months (95% confidence interval, 3–7 months) before disease progression, contented stop, or refusal to continue treatment. It was very difficult to evaluate the effect of adding Rapa + HCQ to metronomic chemotherapy on the PFS in such a heterogeneous group of patients. Nevertheless, the state of 2 patients progressed from PD to PR and that of another 8 patients progressed from SD to PR, following Rapa + HCQ salvage treatment, suggesting an encouraging response to this treatment.

**Table 2:** Disease status and response after addition of Rapa + HCQ to metronomic chemotherapy

Patient #	Tumor type	Performance status	Previous therapy after initial treatment failure	Control metronomic chemotherapy	Control treatment duration (months)	Response to control metronomic chemotherapy	Best response to Rapa + HCQ salvage chemotherapy	Evaluation method	Treatment duration (months)	Current status
1	Prostate ca with bone meta	1	Hormone therapy	Cyproterone 1# bid Docetaxel 40 mg q2wk	4	PD	SD	PSA > 50% decline	6	A
2	Prostate ca with bone meta	1	Hormone therapy	Cyproterone 1# bid Docetaxel 40 mg q2wk	2	PD	SD	PSA > 50%	3	A
3	Prostate ca with LN, bone meta	1	Hormone therapy	Cyproterone 1# bid Docetaxel 40 mg q2wk	3	SD	SD	PSA > 50% decline	3	A
4	Prostate ca with LN meta	0	Hormone therapy	Cyproterone 1# bid Docetaxel 40 mg q2wk	3	SD	PR	RECIST	3	A
5	Bladder ca with lung meta	1	—	Carboplatin 150 mg+ Gemcitabine 1400 mg q2wk	4	PD	SD	RECIST	8	A
6	Bladder ca with liver meta	1	Cisplatin 50 mg/m^2^; D1 +gemcitabine 1000 mg/m^2^ D, 8 q3w	Carboplatin 150 mg+ Gemcitabine 1400 mg q2wk	3	PD	PR	RECIST+ CFR 21.1 decline > 50%	3	A
7	Bladder ca with neck LN meta	0	—	Carboplatin 150 mg+ Gemcitabine 1400 mg q2wk	4	PD	PD	RECIST	3	A
8	Bladder ca with lung meta	1	—	Carboplatin 150 mg+ Gemcitabine 1400 mg q2wk	4	SD	PR	RECIST	8	D
9	Lung ca, adenocarcinoma with brain, pleural meta	1	TKI, RT, Cisplatin+ Pemetrexed	Vinorelbine 30 mg p.o. weekly, Docetaxel 40 mg q2wk	6	PD	PR	RECIST+ CEA decline > 50%	7	A
10	Lung ca, squamous carcinoma with pleural effusion	1	RT, Cisplatin+ Gemcitabine	Vinorelbine 30 mg p.o. weekly, Docetaxel 40 mg q2wk	3	PD	SD	RECIST	3	A
11	Lung ca, adenocarcinoma with multiple meta	1	TKI, RT, Cisplatin+ Pemetrexed	Vinorelbine 30 mg p.o. weekly, Docetaxel 40 mg q2wk	4	PD	SD	RECIST+ CEA decline > 50%	8	A
12	Lung ca, adenocarcinoma with multiple meta	0	TKI, Cisplatin+ Pemetrexed	Vinorelbine 30 mg p.o. weekly, Docetaxel 40 mg q2wk	2	PD	SD	RECIST	4	A
13	Rectal ca with peritoneal seeding	0	FOLFIRI	Capecitabine 1# bid +Irinotecan 100 mg/m^2^ q2wk	4	SD	PR	RECIST	3	A
14	Colon ca with lung and pelvic meta	0	FOLFOX+ Bevacizumab	Capecitabine 1# bid +Irinotecan 100 mg/m^2^ q2wk	5	PD	SD	Tumor marker > 50% decline	4	A
15	Breast ca with liver and abdomen LN meta	1	CAF, Docetaxel	Vinorelbine 30 mg p.o. weekly, Capecitabine 1# bid, Gemcitabine 800 mg/m^2^ q2wk	3	SD	PR	RECIST	4	A
16	Breast ca with bone meta	0	Tamoxifen	Vinorelbine 30 mg p.o. weekly, Capecitabine 1# bid, Gemcitabine 800 mg/m^2^ q2wk	5	PD	SD	Tumor marker > 50% decline	8	A
17	Breast ca with chest wall recurrence	0	CAF, Docetaxel	Vinorelbine 30 mg p.o. weekly, Capecitabine 1# bid, Gemcitabine 800 mg/m^2^ q2wk	4	SD	PR	RECIST	12	A
18	Breast ca with bone and chest wall recurrence	1	RT, CAF, Docetaxel	Vinorelbine 30 mg p.o. weekly, Capecitabine 1# bid, Gemcitabine 800 mg/m^2^ q2wk	2	PD	SD	RECIST	5	A
19	Phylloides tumor with lung meta	0	CAF	Cyclophosphamide (50)1# qod, Etoposide(50) 1# qod, Gemcitabine 1400 mg q2wk	3	SD	PR	RECIST	12	D
20	Fibrosarcoma with lung meta	0	—	Cyclophosphamide (50)1# qod, Etoposide(50) 1# qod, Gemcitabine 1400 mg q2wk	3	SD	PR	RECIST	6	D
21	Sarcoma, nonspecific with LN meta	0	—	Cyclophosphamide (50)1# qod, Etoposide(50) 1# qod, Gemcitabine 1400 mg q2wk	2	PD	PD	RECIST	3	D
22	Leiomyosarcoma of uterus with pelvic recurrence	1	—	Cyclophosphamide (50)1# qod, Etoposide(50) 1# qod, Gemcitabine 1400 mg q2wk	2	PD	PD	RECIST	4	D
23	Buccal ca, local recurrence	1	CCRT	Cyclophosphamide 50 mg qd, Methotrexate 50 mg q2wk, Tegafur and Uracil (UFT) 1# tid cisplatin 30/m^2^ q2wk	3	PD	SD	RECIST	6	D
24	Gingival ca, with lung meta	1	—	Cyclophosphamide 50 mg qd, Methotrexate 50 mg q2wk, Tegafur and Uracil (UFT) 1# tid	2	SD	PR	RECIST	8	A
25	Parotid gland ca, adenoid cystic carcinoma with multiple meta	1	Doxorubicin 50/m^2^ + cisplatin 60/m^2^ q3w	Cyclophosphamide 50 mg qd, cisplatin 30/m^2^ q2wkMethotrexate 50 mg q2wk, Tegafur and Uracil (UFT) 1# tid cisplatin 30/m^2^ q2wk	3	PD	PD	RECIST	3	A

Abbreviations: Ca, cancer; meta, metastasis; bid, twice a day; tid, three times a day; qd, every day; qod, every other day; q2wk, every 2 weeks; LN, lymph node(s); CCRT, concomitant chemoradiotherapy; TKI, tyrosine kinase inhibitor; RT, radiotherapy; FOLFIRI, Irinotecan with fluorouracil and folinic acid; FOLFOX, oxaliplatin with fluorouracil and folinic acid; CAF, 5-fluorouracil, doxorubicin, cyclophosphamide.

### Toxicities

Data related to non-hematologic toxicity indicated that therapy was well tolerated. As shown in Table 3, 8 patients (32%) reported grade ≥1 fatigue, including 1 who had grade 3 fatigue and had to discontinue the treatment; diarrhea followed as the second most common toxicity, with 4 (16%) patients exhibiting grade 2 and 1 (4%) reporting grade 3 diarrhea. Two (8%) patients had mucositis and 1 (4%) reported grade 3 nausea/vomiting or renal toxicity. One patient (patient # 23) had grade 3 cardiotoxicity, with a left ventricle ejection fraction of 35%. The patient recovered after discontinuing all treatment. She had no history of doxorubicin usage. One patient (patient # 22) experienced grade V hepatitis, which was attributed to the reactivation of previously unnoted hepatitis B virus. She had not been administered prophylactic anti-viral medicine. Myelotoxicity was relatively common, with 8 patients (32%) developing grade ≥ 3 thrombocytopenia, 6 patients (24%) developing grade ≥ 3 leucopenia and 3 patients (12%) having grade ≥ 3 anemia. None of the patients developed febrile neutropenia, and they all recovered quickly from myelotoxicities after one to two weeks of treatment interruption.

**Table 3:** Chemotherapy toxicity

	Metronomic chemotherapy		Rapa + HCQ + metronomic chemotherapy	
	N (%)		N (%)	
	Grade 3	Grade 4	Grade 3	Grade 4
Leucopenia	3 (12%)	0	6 (24%)	0
Thrombocytopenia	2 (8%)	0	7 (28%)	1 (4%)
Anemia	2 (8%)	0	3 (12%)	0
Nausea/Vomiting	0	0	1 (4%)	0
Mucositis	0	0	1 (4%)	0
Diarrhea	0	0	1 (4%)	0
Asthenia	0	0	1 (4%)	0
Hepatic toxicity	0	0	0	1 (4%)
Renal toxicity	0	0	0	0
Cardiac toxicity	0	0	1 (4%)	0
Skin rash	0	0	0	0
Retinopathy	0	0	0	0

## DISCUSSION

This is the first report on the addition of Rapa and HCQ to conventional metronomic chemotherapy, which was found to be safe and well tolerated in a variety of cancer types. Most importantly, this chemotherapeutic combination was associated with a 40% observed response rate and an 84% disease stabilization in a cohort of patients refractory to their chemotherapy regimen. The significant clinical benefit observed in patients resistant to chemotherapy was unexpected and merits further investigation.

A growing number of clinical studies have shown that the anticancer effect of metronomic chemotherapy is primarily a consequence of its anti-angiogenic effect. Low-dose chemotherapy is preferentially cytotoxic to dividing endothelial cells [30], leading to death of circulating endothelial progenitor cells [31] and a decreased microvessel density [32]. Rapa, although traditionally thought of as an immunosuppressive drug, may also inhibit tumor growth and have anti-angiogenic effects [33]. Recently, CQ has also been reported not only to reduce tumor growth, but also to improve tumor angiogenesis in a mechanism independent of autophagy [34]. CQ normalized tumor vessel structure and perfusion function, improved hypoxia, and reduced tumor invasion through endosomal Notch 1 trafficking and signaling in endothelial cells [34]. The synergistic effect of everolimus and CQ combination on endothelial cell apoptosis was found to be linked to the down-regulation of ERK1/2 phosphorylation in these cells [26]. Clearly, the combination of metronomic chemotherapy, Rapa and HCQ must activate common anti-angiogenic pathways. The CT attenuation of Hounsfield Units in bladder cancer with liver metastases as shown in Figure 1 is typical evidence of anti-angiogenic effects after combination treatment [35].

It is not yet known whether the positive results obtained using this strategy is caused solely by additive anti-angiogenic effects or by modulation of autophagy. Several phase II clinical trials have examined the potential benefits of adding either mTOR inhibitors (Rapa, everolimus, tenolimus) or HCQ (autophagy-lysosome inhibitor) to conventional chemotherapy in the treatment of patients with malignant gliomas [36, 37], non-Hodgkin lymphomas [38], sarcoma [39], melanoma [15, 16], breast [40], non-small cell lung [41, 42], esophageal [43], and head and neck cancers [44]. Neither strategy had resulted in impressive results. This is the first clinical report describing concomitant use of Rapa, HCQ, and chemotherapy in various cancer types. The results of this self-controlled study indicated that such combinations were not only effective, but also could reverse drug resistance. The autophagy inducer and lysosomal inhibitor in combination with chemotherapy seem to work synergistically. The doses of Rapa (2 mg/day) and HCQ (400 mg/day) were derived from conventional therapeutic doses used in rheumatoid arthritis, kidney transplantation, or treatment of lymphangioleiomyomatosis [45-47]. These doses are not MTD, especially for HCQ, which was reportedly used at a dose of 1200 mg/day in combination with temozolomide for the treatment of solid tumors [8]. The dosages of these drugs were not obtained through serial phase I studies of different chemotherapeutic combinations, and therefore, the doses of these drugs could be increased further prior to committing to a phase II trial. Another weakness of this report is the lack of pharmacodynamic assays in tumor tissues or peripheral mononuclear cells. Indeed, the targeted signaling pathways (autophagy or angiogenesis) might not have been modified by these drugs. We have conducted a molecular imaging study in sarcoma patients before and after 2 weeks of treatment with Rapa (2 mg/day) and HCQ (400 mg/day) on the basis of reports that cancer-associated fibroblasts could be potential oncotargets [48].

Although a clinical benefit rate of 84% in this selectively chosen patient cohort seems impressive, additional randomized phase II trials are required before the claim of actual clinical benefits can be accepted. Nevertheless, the real benefit of dual modulation of autophagy might be even more impressive in combination with standard dose chemotherapy.

## MATERIALS AND METHODS

### Patient selection

Patients chosen for analysis were required to have incurable metastatic/recurrent disease and no clinical response from current metronomic chemotherapy regardless of the primary tumor type. Patients needed to continue the same metronomic chemotherapy in addition to Rapa (2 mg qd) and HCQ (400 mg qd) as salvage treatment. They needed to have an Eastern Cooperative Oncology Group performance status of 0 to 1; a life expectancy of at least 3 months before salvage treatment, and either have at least one single site of measurable (two-dimensional) disease or serial and continuous (>3 monthly measurement) elevated tumor markers (at least twice the upper limit) in patients with non-measurable lesions. Patients were excluded if treatment with metronomic therapy and salvage therapy was shorter than 12 weeks, unless there was a radiographic confirmation of disease progression. This retrospective study was approved by our institutional review board (IRB).

### Treatment

Patients were treated using different metronomic regimen based on their disease type: cyproterone, 50 mg orally 1 tablet twice daily (1# bid), and, docetaxel, 40 mg per body intravenously (i.v.) every 2 weeks (q2w) for prostate cancer; capecitabine, 500 mg 1# bid, vinorelbine, 30 mg orally once a week (qw), and gemcitabine, 800 mg/m^2^ i.v. q2w for breast cancer; vinorelbine, 30 mg orally qw plus docetaxel, 40 mg per body i.v. q2w for lung cancer; capecitabine, 500 mg 1# bid, and irinotecan, 100 mg/m^2^ i.v. q2w for colorectal cancer; carboplatin, 150 mg per body, plus gemcitabine, 1400 mg per body i.v. q2w for bladder cancer; cyclophosphamide, 50 mg orally every other day (qod), methotrexate, 50 mg orally qw, tegafur and uracil (UFT), 1# three times a day (tid), and cisplatin, 30 mg/m^2^ i.v. q2w for head and neck cancers; cyclophosphamide, 50 mg orally qod, etoposide, 50 mg orally qod, and gemcitabine, 1400 mg per body i.v. q2w for sarcoma. Rapa (2 mg) and HCQ (400 mg) treatments were started following the physician's suggestion the patients' signing an agreement for the additional treatment. The dose of Rapa, HCQ was choosing from conventional therapeutic doses used in rheumatoid arthritis, kidney transplantation. The metronomic chemotherapy schedule was followed as given above. There was no dose modification of Rapa, HCQ, or chemotherapy. The only treatment interruption was scheduled when grade ≥ 3 myelotoxicity was observed, with a maximum delay of 3 weeks being allowed. Supportive care agents such as anti-emetics, antibiotics, loperamide, growth factors, transfusions, or fluid supply were administered as indicated.

Treatment was discontinued with the development of grade IV non-myelotoxicity, patient intolerance, or disease progression. Treatment-related toxicity was assessed every 2 weeks. Toxicity was scored according to the Common Terminology Criteria for Adverse Events (CTCAE) v4.0 [27]. Response was evaluated using chest radiography, computed tomography (CT), or positron emission tomography-computed tomography, which were obtained in principle every 2 to 3 months. Tumor markers were assessed every 1 to 3 months. Response Evaluation Criteria in Solid Tumor (RECIST) v1.1 guidelines were used as follows: complete response (CR, disappearance of measurable disease without development of new lesions, with tumor markers dropping to normal range), partial response (PR, at least 30% reduction in the sum of the longest diameters measured at disease sites or enhanced area), progressive disease (PD, at least 20% increase in the sum of the longest diameter measured disease sites or appearance of new lesions), and stable disease (SD, if determination did not meet criteria of CR, PR, or PD, or those patients with tumor markers decline of > 50% in non-measurable lesions) [28, 29]. The radiologic evaluation of the response was obtained by consensus following meeting of the three principle investigators (K.H.C., K.L.Y., and H.L.K.). Clinical benefit rate was defined as the percentage of patients without disease progression for more than 3 months.
